# A novel tri-band T-junction impedance-transforming power divider with independent power division ratios

**DOI:** 10.1371/journal.pone.0178956

**Published:** 2017-06-06

**Authors:** Yongle Wu, Yangyang Guan, Zheng Zhuang, Weimin Wang, Yuanan Liu

**Affiliations:** Beijing Key Laboratory of Work Safety Intelligent Monitoring, School of Electronic Engineering, Beijing University of Posts and Telecommunications, Beijing, China; University of Calgary, CANADA

## Abstract

In this paper, a novel *L* network (LN) is presented, which is composed of a frequency-selected section (FSS) and a middle stub (MS). Based on the proposed LN, a tri-band T-junction power divider (TTPD) with impedance transformation and independent power division ratios is designed. Moreover, the closed-form design theory of the TTPD is derived based on the transmission line theory and circuit theory. Finally, a microstrip prototype of the TTPD is simulated, fabricated, and measured. The design is for three arbitrarily chosen frequencies, 1 GHz, 1.6 GHz, and 2.35 GHz with the independent power division ratios of 0.5, 0.7, and 0.9. The measured results show that the fabricated prototype is consistent with the simulation, which demonstrates the effectiveness of this proposed design.

## Introduction

In modern wireless communication systems, the ever increasing demand on the high-performance indicators has led to plenty of investigations for radio frequency (RF)/microwave devices. Owing to the indispensability of the power dividers (PDs) and filters in RF/microwave front end devices, vast research has been dedicated to the PDs [1–9] and filters [10–12] with various performances over the past decades. Hereinto, in order to achieve the multi-band concurrent operation, a number of PDs have been reported, which includes arbitrary power division [1, 2, 9], controllable frequency ratio [4, 5], and multi-way transmissions [1, 6].

The combination of the multi-way transmissions and arbitrary power division is acquired by using a two-section dual-frequency transformer in each way [1]. However, its circuit size is very large. In order to further improve the isolated frequency band, a dual-band Gysel power divider (PD) is reported [2] based on two Schiffman phase shifters. In addition, dual-band PDs with controllable frequency ratio are designed by using the lumped elements [3] and the composite right- and left-handed transmission lines [4], respectively. Furthermore, an earlier reported dual-band PD [5] makes a breakthrough in multi-way application with equal power division. However, the aforementioned PDs are only applicable to dual-band application. And it is hard to design for more than two frequencies. Even so, there still exist several tri-band PDs [6–8]. For example, a tri-band PD [6] is implemented based on a three-section transmission line transformer. However, the closed-form formulas for parameters solution have not been obtained and extra optimization is usually required. A novel impedance transformer [7] transforms the derived admittances at three frequency points and completes the design of a tri-band PD. Note that the reported PD in [8] using embedded transversal filtering sections can realize single or multi-band capability, as demonstrated with a quad-band example. Though the multi-band PDs with satisfactory performances have been extensively investigated, independent power division ratios at arbitrary operating frequency have not been achieved. The main limitation of the presented frequency-dependent transformer [9] is the dual-frequency operation. To the best of the authors’ knowledge, there is few reported research on the multi-band PDs with independent power division and impedance-transforming function.

In this paper, an original TTPD with independent power division ratios at arbitrary frequency is proposed. And a novel LN with multi-band application is designed and analyzed in detail, which is smaller in size than the PI-type impedance transformer [13]. The LN utilized in the TTPD fulfills the transformation between the equivalent input impedance and the terminal impedance at each operating frequency. Furthermore, the complete design methodology of the TTPD and the formulas for parameters calculation are presented. For theoretical verification, the ideal TTPD is simulated with the ideal electrical parameters calculated by analytical equations. For experimental verification, the microstrip prototype is fabricated by employing the normal printed circuit board (PCB) fabrication technology. The presented TTPD operating at the center frequency of 1 GHz, 1.6 GHz, and 2.35 GHz with the independent power division ratio of 0.5, 0.7, and 0.9 is designed, simulated and measured. A good agreement between the simulated and measured results is observed, which demonstrates the effectiveness of this design.

## Methods

### Theoretical design and numerical calculation

**Fig 1:**
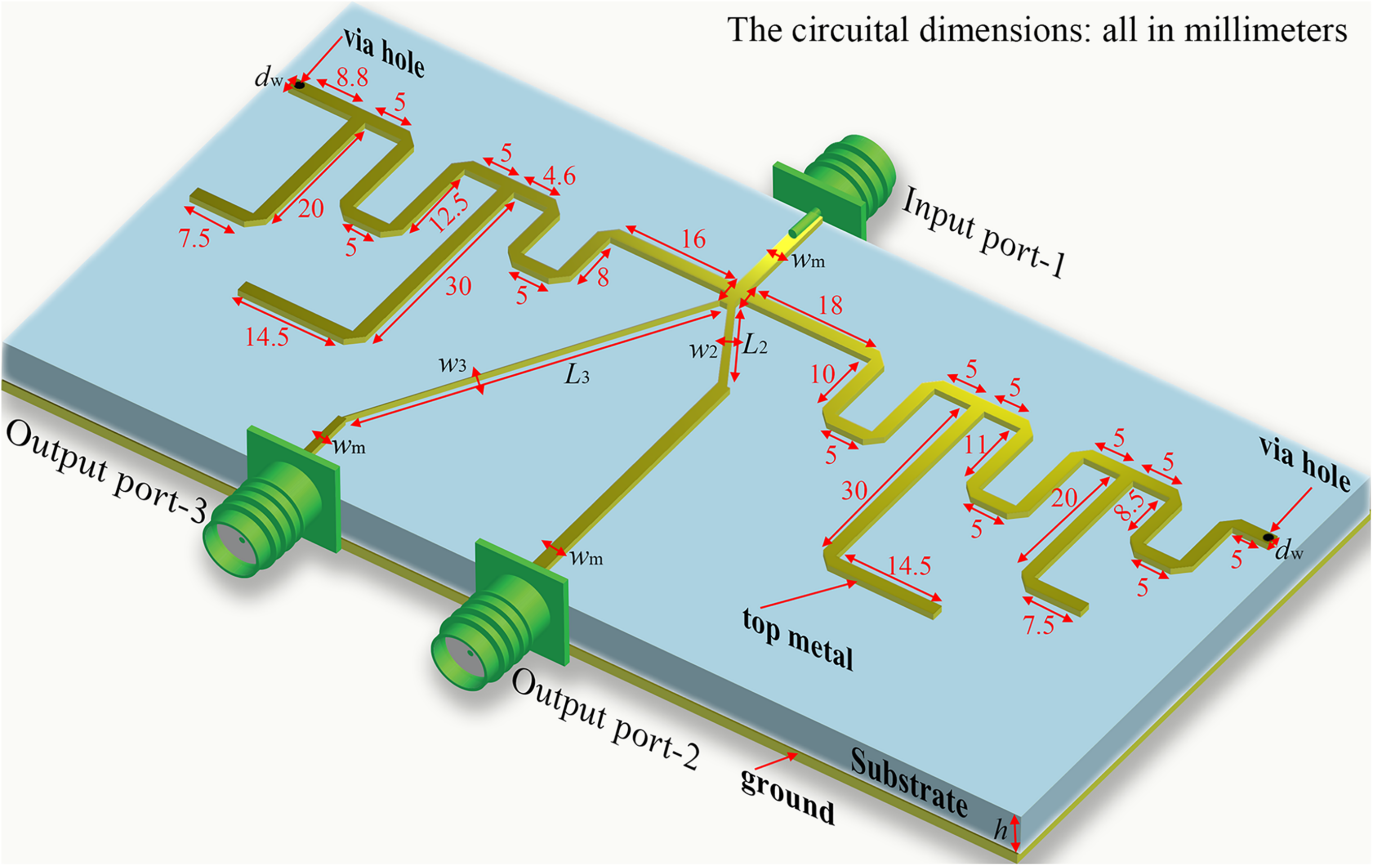
The three-dimensional (3D) structure of the proposed TTPD. This TTPD is excited by 50 Ω microstrip feeding line. The ground is constructed completely by a metal and the applied substrate is the Rogers 4350B with the relative permittivity of 3.48, the thickness *h* of 0.762 mm and the loss tangent of 0.0037. The length of the FSS in output path-2 and output path-3 are marked in the picture, *w*_*m*_ is the width of the 50 Ω microstrip feeding lines and the stubs in the FSS, *w*_2_ and *w*_3_ (*L*_2_ and *L*_3_) are the width (length) of the MS in the two output paths, *d*_*w*_ is the diameter of the via holes. *w*_*m*_ = 1.7 mm, *w*_2_ = 0.9 mm, *w*_3_ = 0.1 mm, *L*_2_ *=* 14.9 mm, *L*_3_ *=* 48.6 mm, *d*_*w*_ = 1.2 mm.

**Fig 2:**
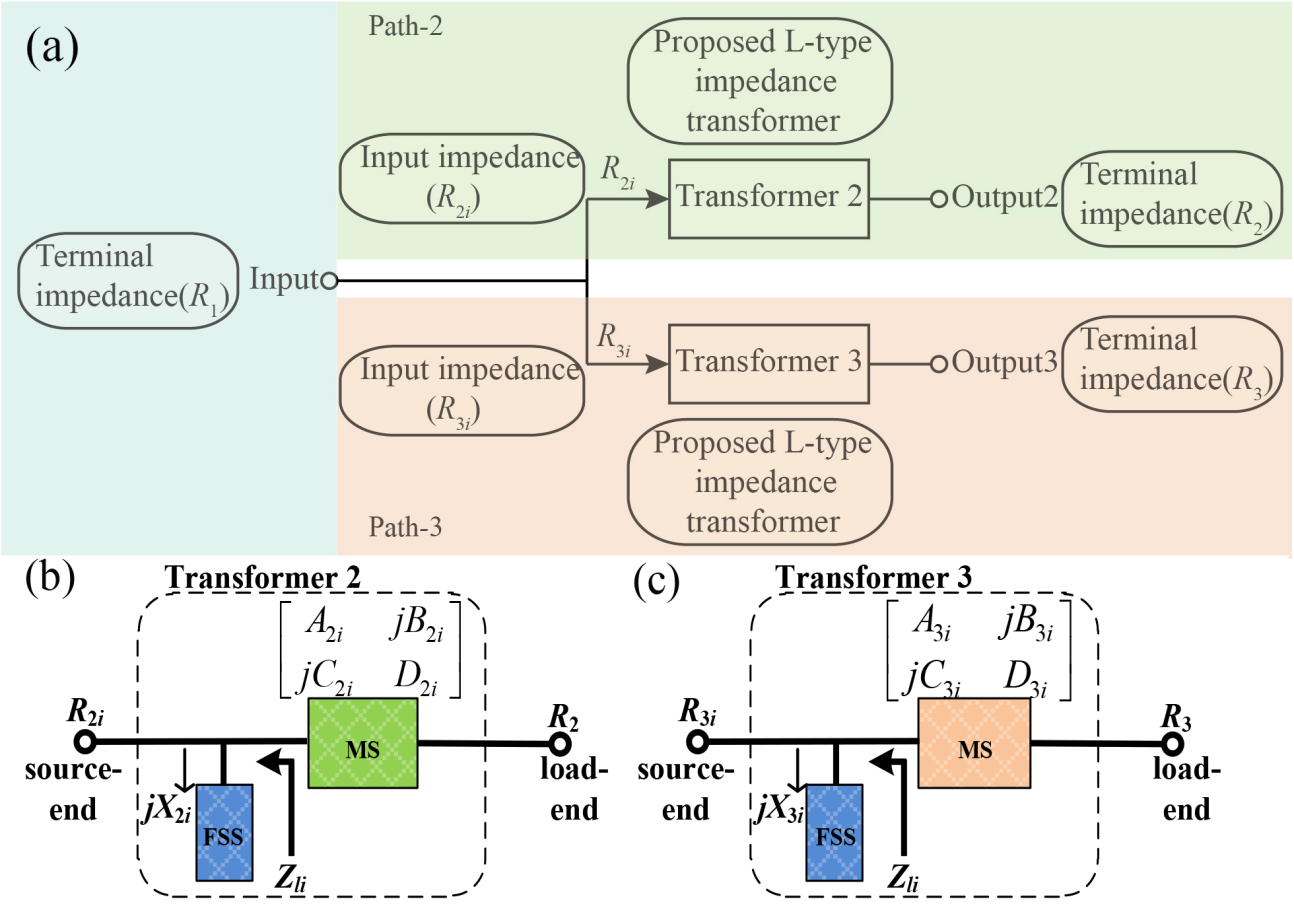
The topology of the TTPD. (a) Each path from the input port to the output port includes the LN shown as Transformer 2 and Transformer 3 in this picture. *R*_1_ (*R*_2_ and *R*_3_) is the terminal impedance of the input (output) port, *R*_2*i*_ and *R*_3*i*_ are the equivalent input impedance calculated in the direction from the input to the output in path-2 and path-3 at each specific frequency *f*_*i*_. The MS and the FSS of the L-type Transformer 2 (Transformer 3) are respectively expressed by its ABCD-matrix and the equivalent input impedance j*X*_2*i*_ (j*X*_3*i*_) in (b) and (c).

Fig 1 reports the three-dimensional (3D) structure of the proposed TTPD and depicts the physical parameters’ definitions and detailed dimensions. The initial dimensions of various elements are found using the equations discussed in the following paragraph. To facilitate a clear analysis, the topology of the TTPD is shown in Fig 2(A) and those of the L-networks used in path-2 and 3 are depicted in Fig 2(B) and 2(C). As shown in (1), the equivalent input impedances *R*_2*i*_ and *R*_3*i*_ in the two output paths and the input terminal impedance *R*_1_ satisfy the theory of parallel circuit and impedance match at each selected frequency *f*_*i*_ (*i =* 1, 2, 3). Moreover, the power division ratio *k*_*i*_, which is the ratio of the output power *P*_2*i*_ and *P*_3*i*_, is related to *R*_2*i*_ and *R*_3*i*_ in (2) for the proposed TTPD [1].

$$\frac{R_{2i}R_{3i}}{R_{2i}+R_{3i}}=R_{1}$$ (1)

$$\frac{R_{2i}}{R_{3i}}=k_{i}^{2}=\frac{P_{3i}}{P_{2i}}$$ (2)

After rearranging (1) and (2), the equivalent input impedance *R*_2*i*_ and *R*_3*i*_ can be expressed in terms of the input terminal impedance and the power division ratio, as given by (3) and (4). That is, *R*_2*i*_ and *R*_3*i*_ are determined once *R*_1_ and *k*_*i*_ are arbitrarily supposed.

$$R_{2i}=R_{1}(1+k_{i}^{2})$$ (3)

$$R_{3i}=R_{1}(1+\frac{1}{k_{i}^{2}})$$ (4)

As shown in Fig 2, the subscripts *S* and *L* uniformly represent the meaning of source-end and load-end. The subscripts *a* and *i* represent the specific output path (*a* = 2, 3) and the specific frequency *f*_*i*_ (*i* = 1, 2, 3) in the following equations. The LN plays the role of impedance transformation between the equivalent input impedance *R*_*ai*_ (*a* = 2, 3; *i* = 1, 2, 3) and the output terminal impedance *R*_*a*_ (*a* = 2, 3) as shown in Fig 2(A). Moreover, Fig 2(B) and 2(C) provide the schematic of the LN in each output path including the MS and the FSS. The FSS is expressed by its equivalent input impedance j*X*_*ai*_ (*a* = 2, 3; *i* = 1, 2, 3). The MS including a cascaded transmission line is represented by its ABCD-matrix in (5). Hereinto, *Z*_*a*_ is the characteristic impedance of the MS and *E*_*a*_ is the electrical length of the MS at the specific frequency *f*_*i*_ (*i* = 1, 2, 3).

$$\left[\begin{array}{cc} A_{ai} & jB_{ai}\\ jC_{ai} & D_{ai} \end{array}\right]=\left[\begin{array}{cc} \cos(E_{a}) & jZ_{a}\sin(E_{a})\\ \frac{j\sin(E_{a})}{Z_{a}} & \cos(E_{a}) \end{array}\right]$$ (5)

The equivalent input impedance Z_*li*_ in (6) is calculated in the direction from the left-end of the MS to the source-end and its conjugate expression is given in (7).

$$Z_{li}=\frac{jR_{ai}X_{ai}}{R_{ai}+jX_{ai}}$$ (6)

$$Z_{li}^{*}=\frac{jR_{ai}X_{ai}}{jX_{ai}-R_{ai}}$$ (7)

Eq (8) is enforced for the condition of impedance transformation between the left- and right-side of the MS [14].

$$\frac{jR_{ai}X_{ai}}{jX_{ai}-R_{ai}}=\frac{A_{ai}R_{a}+jB_{ai}}{jC_{ai}R_{a}+D_{ai}}$$ (8)

After rearranging (8) and extracting its real and imaginary parts, the equivalent input impedance j*X*_*ai*_ of the FSS is obtained in (9).

$$X_{ai}=\frac{A_{ai}R_{ai}R_{a}}{C_{ai}R_{ai}R_{a}-B_{ai}}=\frac{B_{ai}R_{ai}}{A_{ai}R_{a}-D_{ai}R_{ai}}$$ (9)

Then, substituting (5) into (9), the electrical parameters *Z*_*a*_ and *E*_*a*_ of the MS are constrained by (10).

$$R^{2}{}_{a}-R_{ai}R_{a}=(R^{2}{}_{a}-Z_{a}^{2})\sin^{2}(E_{a})$$ (10)

In addition, since (10) is derived at arbitrary specific frequency *f*_*i*_ (*i* = 1, 2, 3) corresponding with independent power division ratio and input port impedance. Thus, (10) can be expanded into three equations according to its respective conditions at each frequency for tri-band application. Besides, this general equation for multi-band application has its restrictive conditions for the solution. The existence of the solution is related with the number of equations and variables. Considering the tri-band design, three expanded equations from (10) are to be solved. As a result, an extra variable parameter should be introduced under the premise of two existing variable parameters (*Z*_*a*_ and *E*_*a*_). In this paper, the output terminal impedance *R*_*a*_ is regarded as an additional variable parameter to solve this problem. In addition, the electrical lengths *E*_*ai*_ (*a* = 2, 3; *i* = 1, 2, 3) in the FSS can be calculated by (11), (12) and (13) based on the detailed derivation in [13]. Hereinto, the characteristic impedance of these open- /shorted-stubs are supposed as Z_0_ (= 50 Ω) for ease of calculation.

$$\tan(E_{a1})=\frac{X_{a1}}{Z_{0}}$$ (11)

$$\cot(E_{a2})=\frac{Z_{0}+2X_{a2}tan(f_{2}E_{a1}/f_{1})-Z_{0}tan^{2}(f_{2}E_{a1}/f_{1})}{X_{a2}-Z_{0}tan(f_{2}E_{a1}/f_{1})}$$ (12)

$$\cot(E_{a3})=\frac{\begin{array}{l} \left[-X_{a3}\tan(f_{3}E_{a2}/f_{1})-X_{a3}\tan(f_{3}\times 90/f_{1})-X_{a3}\tan(f_{3}\times 90/f_{2})+Z_{0}\tan(f_{3}E_{a1}/f_{1})\tan(f_{3}\times 90/f_{1}\right)\\ +Z_{0}\tan(f_{3}E_{a1}/f_{1})\tan(f_{3}\times 90/f_{2})+Z_{0}\tan(f_{3}E_{a1}/f_{1})\tan(f_{3}E_{a2}/f_{2})+Z_{0}\tan(f_{3}E_{a2}/f_{2})\tan(f_{3}\times 90/f_{2})\\ +X_{a3}\tan(f_{3}E_{a2}/f_{2})\tan(f_{3}\times 90/f_{1})\tan(f_{3}\times 90/f_{2})+X_{a3}\tan(f_{3}E_{a1}/f_{1})\tan(f_{3}E_{a2}/f_{2})\tan(f_{3}\times 90/f_{2})\\ -Z_{0}\tan(f_{3}E_{a1}/f_{1})\tan(f_{3}E_{a2}/f_{2})\tan(f_{3}\times 90/f_{1})\tan(f_{3}\times 90/f_{2})-Z_{0}-X_{a3}\tan(f_{3}E_{a1}/f_{1})] \end{array}}{\begin{array}{l} \left[-X_{a3}+Z_{0}\tan(f_{3}E_{a1}/f_{1})+Z_{0}\tan(f_{3}E_{a2}/f_{2})+X_{a3}\tan(f_{3}E_{a1}/f_{1})\tan(f_{3}E_{a2}/f_{2}\right)\\ +X_{a3}\tan(f_{3}E_{a2}/f_{2})\tan(f_{3}\times 90/f_{1})-Z_{0}\tan(f_{3}E_{a1}/f_{1})\tan(f_{3}E_{a2}/f_{2})\tan(f_{3}\times 90/f_{1})] \end{array}}$$ (13)

Finally, the design steps of the proposed TTPD in this paper are summarized as follows:

1. Determine the three frequency points *f*_*i*_ (*i* = 1, 2, 3) as 1 GHz, 1.6 GHz, and 2.35 GHz with the corresponding power division ratios *k*_*i*_ (*i* = 1, 2, 3) of 0.5, 0.7, and 0.9 in (2).
2. Determine the input port impedance *R*_1_ (= 50 Ω) and calculate the respective equivalent input impedance *R*_*ai*_ (*a* = 2, 3; *i* = 1, 2, 3) at each frequency *f*_*i*_ in each output path by (3) and (4). Then, subsequently solve the electrical parameters *Z*_*a*_ and *E*_*a*_ (*a* = 2, 3) of the MS and the additional variable *R*_*a*_ (*a* = 2, 3) by using the three expanded equations from (10).
3. Calculate the equivalent input impedance j*X*_*ai*_ (*a* = 2, 3; *i* = 1, 2, 3) in the FSS by (8) and (9). Then, assume the characteristic impedance of these stubs in the FSS is *Z*_0_ (= 50 Ω) for ease of calculation. Finally, determine the electrical length *E*_*ai*_ (*a* = 2, 3; *i* = 1, 2, 3) of these subs by (11), (12) and (13).

**Fig 3:**
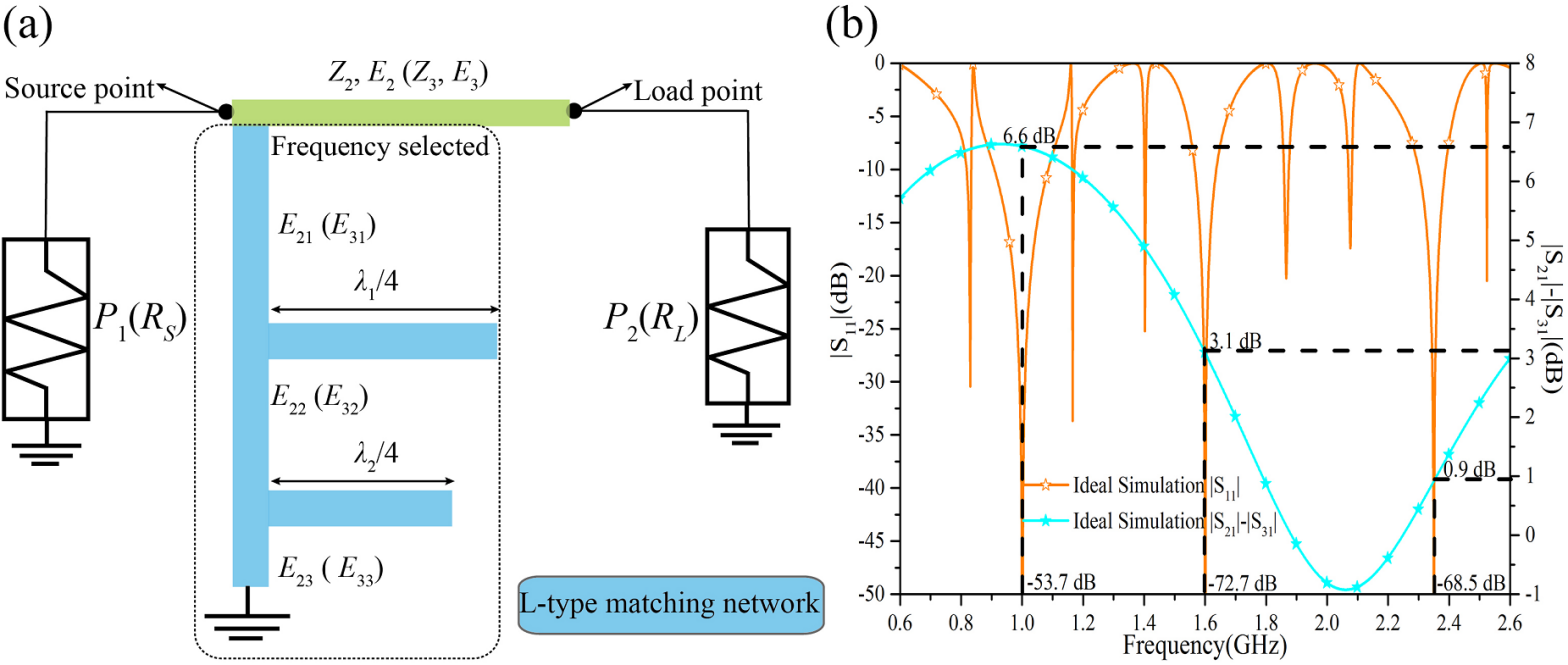
The layout of the proposed LN and the ideal simulation results of the TTPD. (a) The LN is made up of six transmission lines with its ideal electrical parameters. In output path-2: *Z*_2_ = 69.83 Ω, *E*_2_ = 28.7 @ 1 GHz, *E*_21_ = 103.2 @ 1 GHz, *E*_22_ = 141.4 @ 1.6 GHz, *E*_23_ = 178.5 @ 2.35 GHz. In output path-3: *Z*_3_ = 138.2 Ω, *E*_3_ = 88.07 @ 1 GHz, *E*_31_ = 90.3 @ 1 GHz, *E*_32_ = 138.4 @ 1.6 GHz, *E*_33_ = 41.8 @ 2.35 GHz. The input terminal impedance *R*_1_ = 50 Ω and the output terminal impedances *R*_2_ = 50 Ω, *R*_3_ = 68.6 Ω. λ_1_ and λ_2_ are the wavelengths of 1 GHz and 1.6 GHz. (b) The simulation results of the TTPD with the ideal electrical parameters in (a).

**Fig 4:**
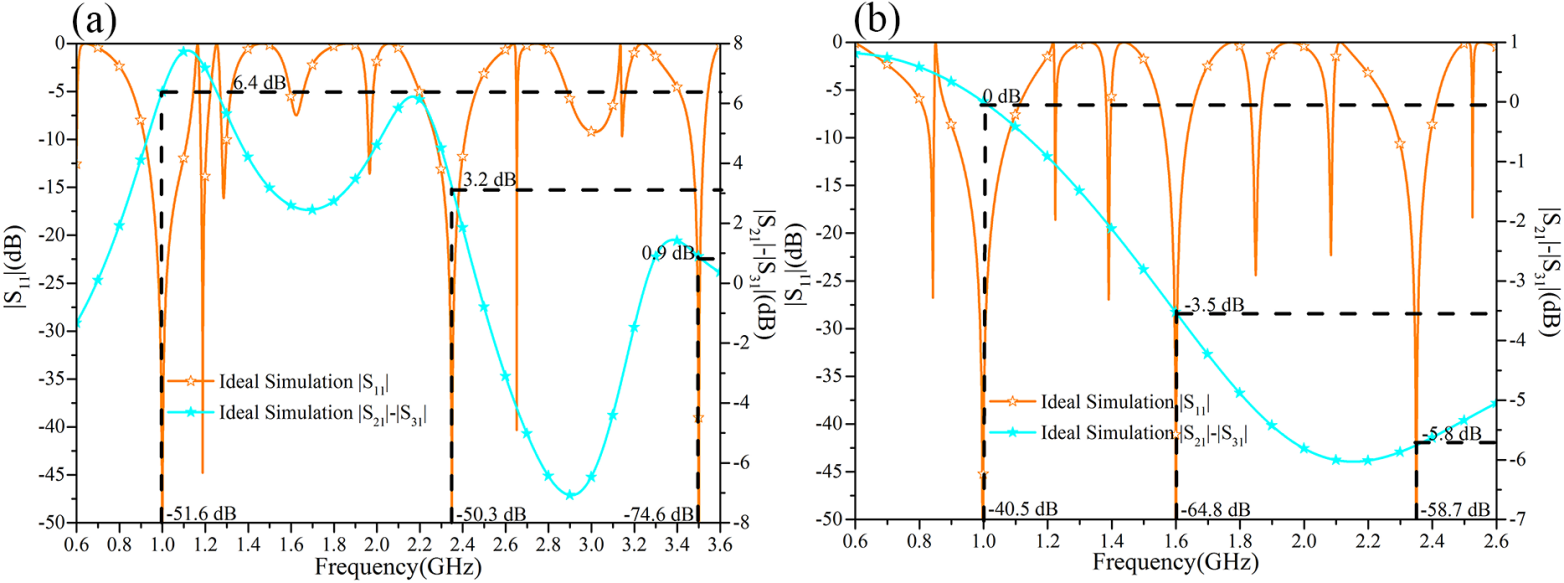
The ideal simulation results of the two comparative examples of the TTPD. (a) Comparative example with same power division ratio and different operating frequency (1 GHz, 2.35 GHz, 3.5 GHz). (b) Comparative example with different power division ratio (0 dB, -3.5 dB, -5.8 dB) and same operating frequency.

According to the above steps, Fig 3(A) shows the layout and the calculated ideal electrical parameters of each stub in the output path-2 and path-3. Generally, the *E*_*a*1_ (*a* = 2, 3) influences three frequency points *f*_*i*_ (*i* = 1, 2, 3), the *E*_*a*2_ (*a* = 2, 3) impacts two frequencies *f*_*i*_ (*i* = 2, 3) and the *E*_*a*3_ (*a* = 2, 3) effects only one frequency *f*_*i*_ (*i* = 3). In Fig 3(B), the difference between the output powers at the corresponding frequency of 1 GHz, 1.6 GHz, and 2.35 GHz are 6.6 dB, 3.1 dB, and 0.9 dB, respectively. The tri-band performance of the TTPD with these calculated values are clearly demonstrated. Moreover, two comparative examples at different frequencies with different power division ratios are simulated with ideal electrical parameters. Fig 4(A) illustrates the comparative example including the approximate same power division ratio at different frequencies (1.0 GHz, 2.35 GHz, 3.5 GHz) comparing with the simulation in Fig 3(B). Next, considering different power division ratios (0 dB, -3.5 dB, -5.8 dB) but keeping the same operating frequencies, another example with its ideal simulated results in Fig 4(B) is shown. Fig 3(B) together with Fig 4 proves the feasibility of the arbitrary three frequencies and the arbitrary independent power division ratios of this design method.

## Results

**Fig 5:**
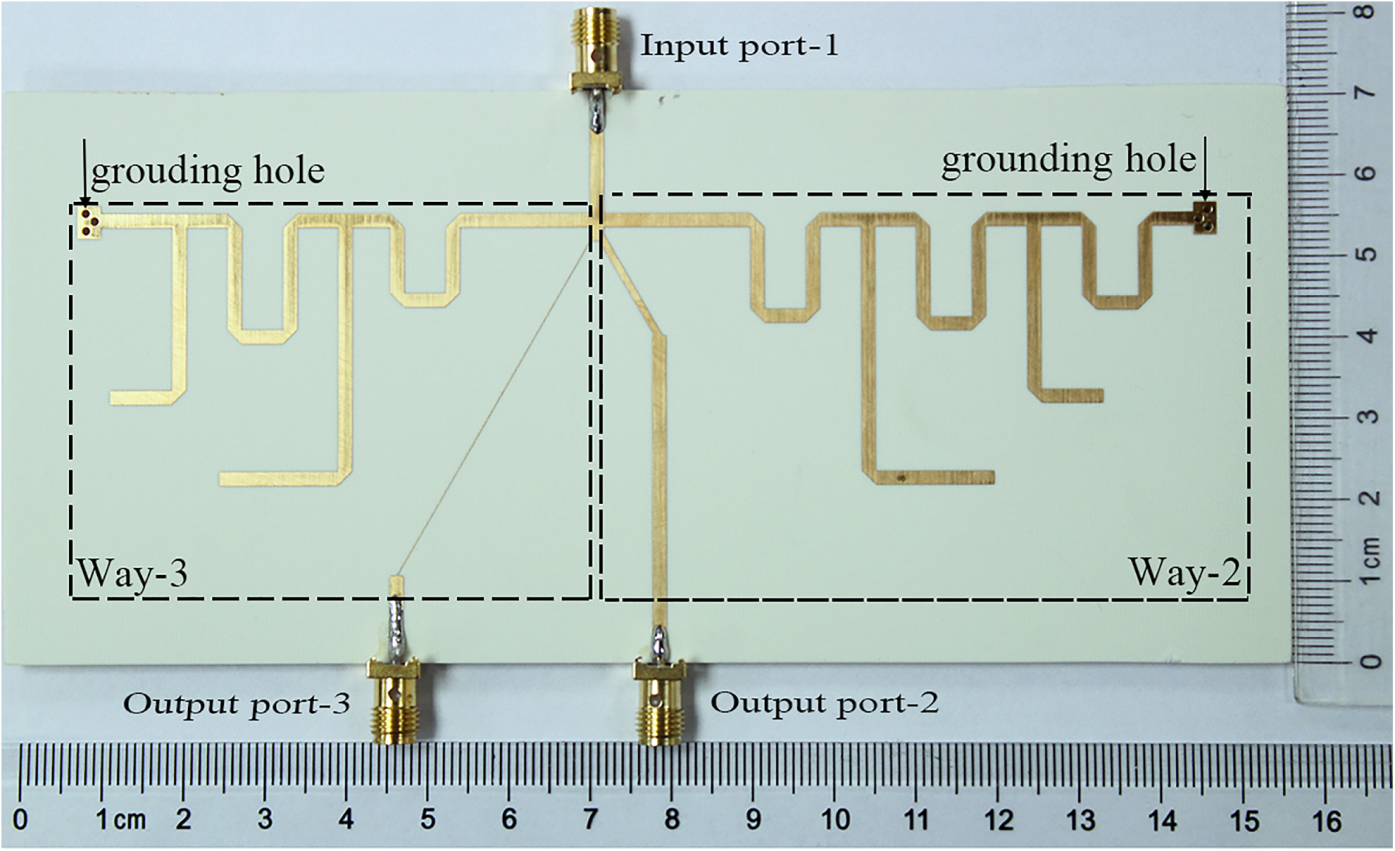
The picture of the TTPD on PCB. The photo of the TTPD is on PCB with the approximate size of 70x150 mm^2^.

To prove the above theory, the proposed TTPD is simulated, fabricated, and measured for verification of the effectiveness. The prototype circuit is built on a Rogers 4350B substrate with a relative permittivity of 3.48, a thickness of 0.762 mm, and a loss tangent of 0.0037. The electromagnetic simulation was done by Advanced Design System (ADS). The electrical parameters of the prototype are given in Fig 3 and the physical dimensions of the fabricated circuit are depicted in Fig 1. Finally, the TTPD with the LN is fabricated by employing the normal PCB fabrication technology for experimental verification. The photo of the fabricated circuit on PCB is shown in Fig 5. The whole dimension of the prototype is approximately 70x150 mm^2^.

**Fig 6:**
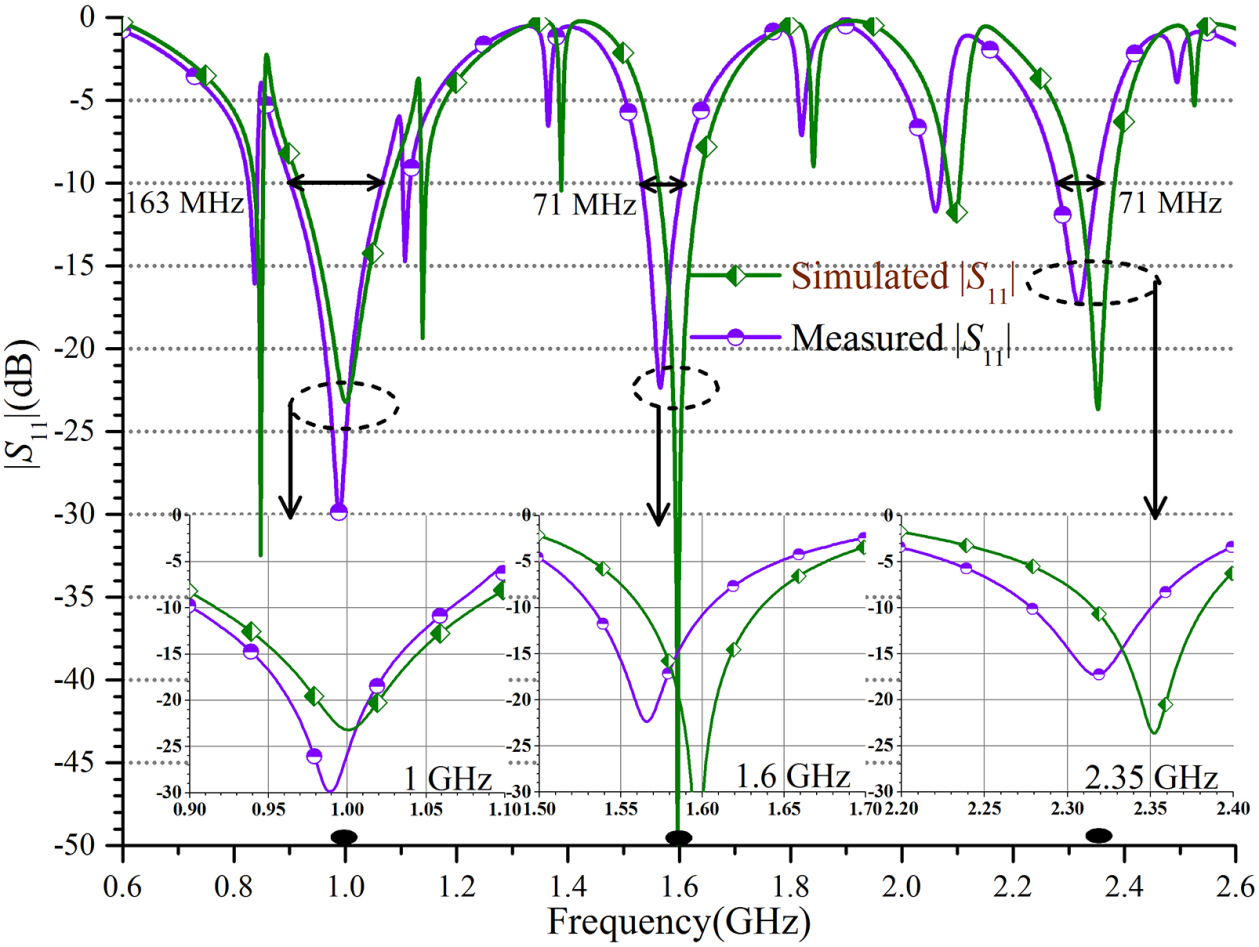
The TTPD with the complete tri-band performance. The simulated and measured input reflection coefficient |*S*_11_| and the three sub-graphs extracted at 1 GHz, 1.6 GHz, and 2.35 GHz are given.

Then, the prototype was measured using the vector network analyzer (E5071A). The simulated and measured input reflection coefficient |*S*_11_| are displayed in Fig 6. The three sub-graphs in Fig 6 are extracted at the corresponding three operating frequencies 1 GHz, 1.6 GHz, and 2.35 GHz. Fig 6 illustrates that there is a good agreement between the simulated and measured results. Besides, the tolerable deviation of less than 5% is observed because of the manufacture error, via holes and the degradation of the substrate and the ordinary SMA (Sub-Miniature version A) connectors. The TTPD operates at the center frequency of 0.98 GHz, 1.57 GHz, and 2.31 GHz and the bandwidth in accordance with |S11|≤-10 dB is about 163 MHz, 71 MHz, and 71 MHz from the measured results. The shift of the center frequency is due to the assembly and fabrication errors and within the allowable range of 5%.

**Fig 7:**
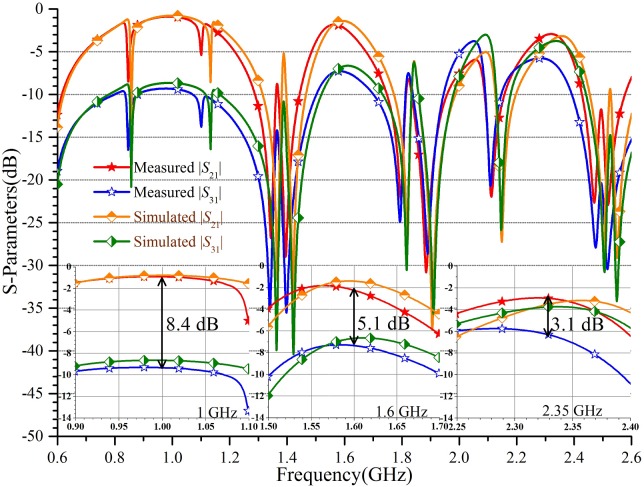
The insertion loss responses of the TTPD. The simulated and measured coefficient |*S*_21_|, |*S*_31_| and the difference of |*S*_21_|-|*S*_31_| are given. The three sub-graphs extracted at three frequency points are given.

Furthermore, Fig 7 reveals the insertion loss responses of the simulated and measured |*S*_21_| and |*S*_31_|, which are -0.9 dB and -9.3 dB, -2.2 dB and -7.3 dB, -2.9 dB and -6.0 dB at the three measured frequency of 1 GHz, 1.6 GHz, and 2.31 GHz, respectively. As shown in Fig 7, the three measured amplitude imbalance of |*S*_21_|-|*S*_31_| are 8.4 dB, 5.1 dB, and 3.1 dB, which is consistent with the expectation. However, a few minor deviations from the design goals are observed. The difference between the simulated and measured results is most likely attributed to the fabrication and assembly errors.

**Fig 8:**
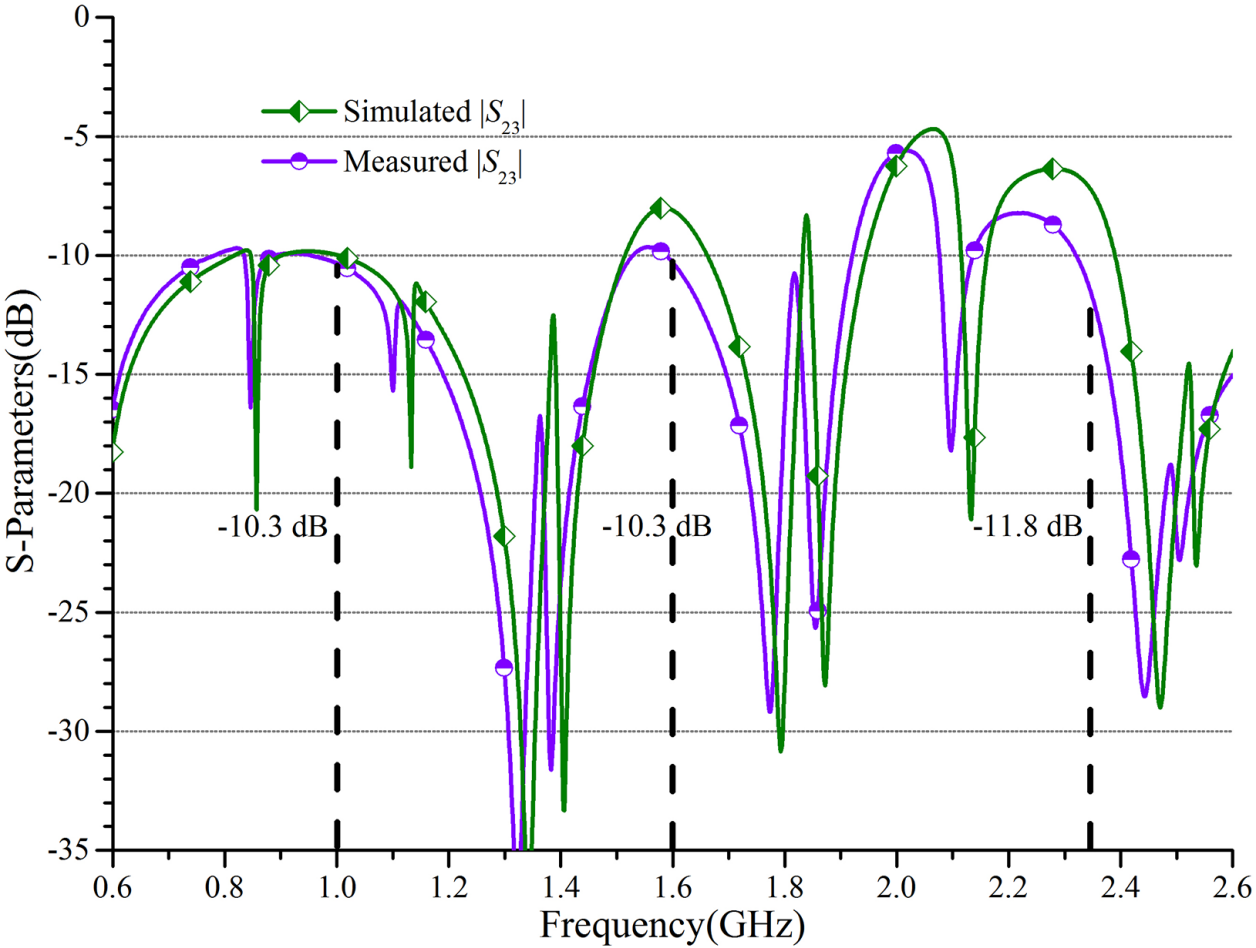
The isolation responses of the TTPD. The simulated and measured coefficient |*S*_23_| are presented.

Moreover, the simulated and measured |*S*_23_| are plotted in Fig 8 to show the isolation response of the PD. It can be found that the basic isolation is obtained without any added isolation resistor. The measured |*S*_23_| are -10.3 dB, -10.3 dB, and -11.8 dB respectively at 1 GHz, 1.6 GHz, and 2.35 GHz. Table 1 compares the proposed TTPD with previous multi-band PDs. It indicates the multi-function design in tri-band operation, impedance transformation, and independent power division ratios of this work.

**Table 1:** Comparison of proposed power divider with existing ones.

Items	Operating Bands	Central Freqs. (GHz)	Method	Power Division Ratio (dB)
[3]	Dual Bands	0.5/2.0	Series LC Network	Equal
[4]	Dual Bands	2.4/3.5	Composite Right- and Left-Handed Transmission Lines	Equal
[6]	Triple Bands	0.9/1.17/2.43	Three-section Transmission Lines	Equal
[7]	Triple Bands	1.0/2.55/3.0	Transmission Lines Network	Equal
[8]	Quad Bands	1.24/2.43/3.54/4.63	Embedded Transversal Filtering Section	Equal
[2]	Dual Bands	0.87/2.13	Two Schiffman Phase Shifter	Equal
[9]	Dual Bands	1.0/2.4	Four-section Transmission Lines	1.5/2.0
Proposed	Triple Bands	1.0/1.6/2.35	L-type Open- and Shorted-stubs	6.6/3.1/0.9

## Discussion

In this paper, a new structure and a complete design method for a TTPD with independent power division ratios at arbitrary frequency are investigated and demonstrated systematically. The main features of this PD including: 1) arbitrary tri-band application; 2) independent power division ratios; 3) simple calculation equations; 4) easy design procedures; 5) convenient implementation using microstrip lines. It should be noted that the power division ratio and the frequency ratio would be constrained for meeting the processing requirements in practical fabrication. Therefore, the proper substrate needs to be selected. For example, the thick or thin substrates are more suitable for large or small characteristic impedances, respectively. Under normal conditions, the ideal parameters can be calculated on the basis of the derived formulas. In this paper, the TTPD with independent power division ratios of 0.5, 0.7, and 0.9 are designed at the three frequency points of 1.0 GHz, 1.6 GHz, and 2.35 GHz. The measured frequency bandwidth of 10-dB return loss is about 163 MHz, 71 MHz, and 71 MHz, respectively. And the measured amplitude imbalance of the output ports are 8.4 dB, 5.1 dB, and 3.1 dB at the three measured center frequency of 0.98 GHz, 1.57, GHz and 2.31 GHz. The isolation responses are -10.3 dB, -10.3 dB, and -11.8 dB respectively at 1 GHz, 1.6 GHz, and 2.35 GHz. It also satisfies the basic isolation without any added isolation resistor. It can be seen that the low return loss, independent power division, tri-band performance, and basic isolation can be obtained simultaneously. Additionally, simple calculation, easy design procedures, convenient implementation, and small size make this PD competitive in practical applications.
